# The protective nasal boosting of a triple-RBD subunit vaccine against SARS-CoV-2 following inactivated virus vaccination

**DOI:** 10.1038/s41392-023-01421-8

**Published:** 2023-04-10

**Authors:** Jingyi Yang, Mei-Qin Liu, Lin Liu, Xian Li, Mengxin Xu, Haofeng Lin, Min Li, Huimin Yan, Yao-Qing Chen, Zheng-Li Shi

**Affiliations:** 1 Vaccine and Immunology Research Center, Translational Medical Research Institute, Shanghai Public Health Clinical Center, Fudan University, Shanghai, 201508 China; 2 Wuhan Institute of Virology, Chinese Academy of Sciences, Wuhan, 430071 China; 3 School of Public Health (Shenzhen), Shenzhen Campus of Sun Yat-sen University, Sun Yat-sen University, Shenzhen, Guangdong 518107 China

**Keywords:** Infectious diseases, Translational research

**Dear Editor**,

Though COVID-19 vaccines have been developed and clinically deployed rapidly, new variants of concern (VOCs) are still emerging frequently and escalating around the world. More breakthrough infections occurred even vaccination rates are high. For possible ending of the pandemic, curbing infection and stopping transmission are priority. Booster approach with either mRNA or inactivated vaccines can reduce COVID-19 severity,^1–3^ but shows limited efficacy against infection and transmission. One of the most important reasons is that serum IgG is hard accessible to mucosal surface of the upper respiratory tract, where the initial infection and replication of SARS-CoV-2 occur.^4,5^ With ability to generate mucosal immunity against SARS-CoV-2, intranasal immunization has attracted worldwide attention.^6^ A most recent report that a combination of systemic mRNA vaccination plus mucosal adenovirus-S immunization induced strong neutralizing antibody responses suggest mucosal booster vaccination is essential to establish robust sterilizing immunity in the respiratory tract against SARS-CoV-2.^7^ Recently, an inhaled COVID vaccine named Convidecia Air has been approved for emergency use as a booster in China.^8^ We have designed a bivalent chimeric triple-RBD immunogen containing one Delta RBD and two Omicron RBDs (3Ro-NC)^9^ (Supplementary Fig. 1) and demonstrated that intranasal (i.n) immunization with 3Ro-NC plus recombinant flagellin KFD adjuvant could induce robust systemic and mucosal immunity against SARR-CoV-2 VOCs. It was noted that the mucosal immunity induced by 3Ro-NC plus KFD adjuvant inhibited Omicron infections in both upper and lower respiratory tracts.

As of September 22, 2022, nearly half of the 12.7 billion COVID-19 vaccines inoculated worldwide are the inactivated SARS-CoV-2 vaccine (IAV) (https://ourworldindata.org/ & https://www.worldometers.info/coronavirus/#countries). However, vaccination with IAV induced a minimal mucosal secretory IgA response in individuals. There is an urgent need to develop a boosting strategy to elicit higher and broad mucosal immune responses for the enormous vaccinees to prevent potential breakthrough infection.

To test the sequential immunization strategy, human ACE2 transgenic mice were immunized with 2-dose IAVs followed by intranasal boost with 2-dose 3Ro-NC plus KFD (***i.n boost group***) or intramuscular boost with 1-dose IAV (***IAV boost group***). Meanwhile, unimmunized mice (***saline group***) and 2-dose IAV immunized mice without boost were used as controls (Fig. 1a). The booster administration increased RBD-binding IgG and neutralizing capacity (Fig. 1b). Consistent with several reports, three-dose COVID-19 vaccines administration are less neutralizing against Omicron variant.^10^ It is of great interest that i.n boost of 2-dose 3Ro-NC plus KFD can improve the breadth and strength of neutralizing ability against Gamma, Omicron BA.1 and BA.2 strains, in contrast to the IAV boost (i.m) (Fig. 1b and Supplementary Fig. 2). As expected, only the ***i.n boost group*** induced SARS-CoV-2-specific mucosal immunity, as evidenced by RBD-specific IgA antibody detected in saliva and virginal lavage fluid (Fig. 1c). Consistently, the neutralizing titers in representative mucosal sample, saliva of ***i.n boost group*** was significantly higher than that in the ***IAV boost group*** (Supplementary Fig. 3)*.* Moreover, ***i.n boost group*** showed comparable RBD-specific IgA antibody titers with that induced by 3-dose of 3Ro-NC + KFD ***i.n*** group, higher than 2-dose of 3Ro-NC + KFD ***i.n*** group (Supplementary Fig. 4).

**Fig. 1:**
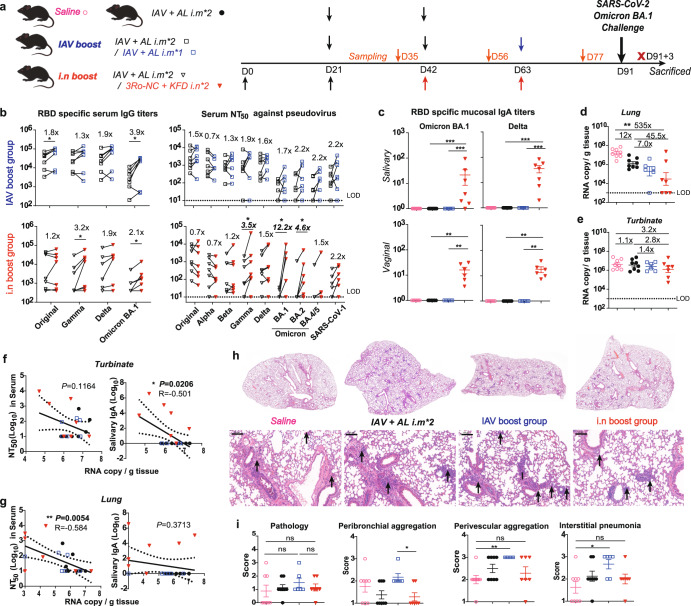
Protection efficacy of boosts against SARS-CoV-2 variant Omicron infection. **a** Diagram scheme of immunization and virus challenge. Briefly, 12–16 weeks old hACE2 transgenic mice were divided into 4 groups: unimmunized saline (8 mice), 2-dose IAVs immunized (8 mice), 2-dose IAVs immunized followed by intranasal boost with 2-dose 3Ro-NC plus KFD (***i.n boost group***, 7 mice) or intramuscular boost with 1-dose IAV (***IAV boost group***, 6 mice). At day 91, all mice were challenged by the SARS-CoV-2 BA.1 strain and sacrificed at day 3 post infection. **b** RBD-specific serum IgG and neutralization antibody titers of serum against pseudo-typed virus, before and post boost. **c** Salivary and vaginal IgA post last immunization. **d**, **e** qPCR tested RNA copies of SARS-CoV-2 RBD in lung and turbinate. **f**, **g** Correlation of RNA copies with Omicron BA.1 RBD-specific salivary IgA and neutralizing titers (dotted lines, 95% confidence interval). **h** Lung sections stained by Hematoxylin and eosin (H&E) (Arrow, inflammatory cell infiltration; Scale bars, 100 µm). **i** Pathological scores, infiltration scores of immunocyte aggregation around bronchioles, pulmonary vessels, and interstitial pneumonia according to the H&E-stained sections. Data are represented as mean ± SEM. Groups were compared using paired *t* test (**b**), or one-way ANOVA (**c**–**e** and **i**). ns not significant; *P < 0.05; **P < 0.01; ***P < 0.001. LOD limited of detection

To investigate effectiveness of the administration of a booster dose, the mice were then intranasally challenged with Omicron BA.1 (5 × 10^4^ TCID_50_ in 50 µl) at 28 days post the last administration. Viral loads, pathological changes and neutralizing antibodies were examined at day 3 post infection. As viral loads are mostly under the detection limit by plaque assay (Supplementary Fig. 5), viral copies were adopted to evaluate the protection against virus infection. Compared to the non-boosted group, reduction of viral copies in the lung of ***i.n boost group*** and ***IAV boost group*** were 45.5 folds and 7 folds respectively, showing a noticeable drop in the ***i.n boost group*** (Fig. 1d). The viral RNA copies in the ***i.n boost group*** have dropped at least 535-fold lower compared to the saline control group. Importantly, 3 of 7 mice showed totally blocking the virus infection in the ***i.n boost group***, which means the intranasal strategy can curb infection (Fig. 1d). To test whether the mucosal immunity can offer protection in upper respiratory tracts, turbinate tissues were taken and measured via qPCR. Intranasal boost group showed the lowest viral titer in nasal turbinate tissue compared to other groups (Fig. 1e). The correlation analysis showed viral RNA copy numbers in the nasal turbinate tissue were negatively correlated with RBD-specific salivary IgA titers, but independent of RBD-neutralizing titers in serum (Fig. 1f). Low viral RNA copy number have been detected in turbinate, indicating that the mucosal immunity conferred protection in the upper respiratory tract. In lung tissue, the viral RNA copy number was negatively correlated with serum neutralizing antibodies titers but not with RBD-specific salivary IgA titers (Fig. 1g). These indicated that the neutralizing antibody responses in serum provided protection in the lower respiratory tract, and the intranasal boosted neutralizing antibody in mucosal tissue conferred protection in the upper respiratory tract. Meanwhile, histopathological examination was performed to analyze infection and immunization related inflammation in the lungs (Fig. 1h and i). It was appreciated that in all four challenged groups, no severe pathology was observed in lungs. But inflammatory cell infiltration around perivascular sites can be observed in the IAV primed mice. In contrast to the IAV boost, the ***i.n*** boost didn’t elevate the level of inflammatory cell infiltration.

In conclusion, despite the significant individual bias in the hACE2 mice, it is still obviously that sequential intranasal immunization with 3Ro-NC plus KFD adjuvant can induce mucosal immunity in the respiratory tract and enhance broad-neutralizing activity against more VOCs. By restriction of initial infection and early replication round of SARS-CoV-2,^4^ the nasal vaccine booster might reduce the risk of secondary transmission to lung to result in severe lung disease as well as hidden transmission to other persons. Combined with property of highly safe and self-administered potential warrant its further clinical trials in humans.

## Supplementary information

supplemental file

## Data Availability

All data used to draw conclusions in the paper are available upon request. The request of experimental materials should be addressed to Z-L.S. (zlshi@wh.iov.cn) (for virological experimental materials) or H.Y. (yanhuimin@shphc.org.cn) (for immunization experimental materials).
