# Supplementary material for: The protective nasal boosting of a triple-RBD subunit vaccine against SARS-CoV-2 following inactivated virus vaccination
Source: Signal Transduct Target Ther. 2023 Apr 10;8:151. doi: 10.1038/s41392-023-01421-8 (PMC10086003; doi:10.1038/s41392-023-01421-8)
Supplement: Supplementary file 1 — supplemental file [file 41392_2023_1421_MOESM1_ESM.docx]

Supplementary Materials for

**The protective nasal boosting of a triple-RBD subunit vaccine against SARS-CoV-2 following inactivated virus vaccination**

Jingyi Yang^1,*^, Mei-Qin Liu^2,*^, Lin Liu^3,*^, Xian Li^1,2*^, Mengxin Xu^3^, Haofeng Lin^2^, Min Li^1^, Huimin Yan^1, †^, Yao-Qing Chen^3,†^, Zheng-Li Shi^2,†^

Correspondence to: zlshi@wh.iov.cn (Z-L.S.); chenyaoqing@mail.sysu.edu.cn (Y-Q.C.); yanhuimin@shphc.org.cn (H.Y.)

**This PDF file includes:**

Materials and Methods

Figures S1 to S5

**Materials and Methods**

**Virus and Cells**

The SARS‐CoV‐2 Omicron strain BA.1 (IVCAS6.7600) was obtained from the National Virus Resource Center (Wuhan, China). This virus strain was propagated in Vero-E6 cells (ATCC CRL-1586). Viral titrations were performed with 10-fold serial dilutions in Vero E6 cells. Two or three days after inoculation, cytopathic effect was scored. Reed-Muench formula was used to calculate the TCID_50_.

**Mice**

The HFH4-human ACE2 transgenic (hACE2) mice on C57BL/6 background were originally obtained from Dr. Ralph Baric (University of North Carolina at Chapel Hill). These hACE2 mice were bred and housed all in individually ventilated cages (IVCs), under speciﬁc pathogen-free (SPF) environment at the Animal Center in Wuhan Institute of Virology (WIV), Chinese Academy of Science (CAS).

**Recombinant Proteins**

Recombinant proteins KFD, 3Ro-NC, RBDs (aa. 319-527) of original strain, Gamma variant, Delta variant and Omicron BA.1 variant were constructed and generated as previously described ^1^. Briefly, KFD which contains D0 and D1 regions of the *E. coli* K12 strain MG1655 flagellin, transformed into the *E. coli* BL21 DE3 strain and induce with 0.1mM IPTG plus 5 mM lactose in Luria–Bertani broth containing 50 μg/ml kanamycin, harvested after incubation at 22 °C for 18 h.

3Ro-NC and RBDs (aa. 319-527) of original strain, Gamma variant, Delta variant and Omicron BA.1 variant were all codon optimized for expression in mammalian cells and cloned into the pcDNA 3.1 plasmid vectors respectively, and transiently expressed in the HEK293F cells by PEI-mediated transfection of the generated plasmids. After grown at 37℃ with 5% CO_2_ for 3 days, cell culture supernatants were harvested.

The his-tag labeled recombinant proteins were puriﬁed on Ni-NTA columns (QIAGEN). After removed lipopolysaccharide (LPS) contamination in the purified KFD and 3Ro-NC, the Limulus assay (Associates of Cape Cod) was utilized to determine residual LPS content, which should be less than 0.05 EU/μg protein for animal experiments.

**Inactivated SARS-CoV-2 vaccine (IAV)**

The inactivated SARS-CoV-2 Original strain virus (IAV) was prepared as previously described ^1^. Briefly, when cytopathic effect (CPE) was shown on day 3 or day 4, the supernatant of Vero cells was harvested. Then β-propiolactone was added 1:4000 (v/v) at 2℃–8℃ for 48 h to inactivate the virus. After cell debris clarification, ultrafiltration, gel-chromatography, ion-exchange chromatography and sterile filtration, the purified inactivated viral particles were formulated.

**Vaccination**

Before the performance of animal experiment, hACE2 mice were randomly assigned. At the prime stage, hACE2 mice in 12-16 weeks old were intramuscularly immunized in the lower hind limb, with 200μg Imject^TM^ Alum adjuvant (Thermo Fisher) (AL-adjuvant) and IAV two times. At the boost stage, the mice were intramuscularly immunized with AL-adjuvant plus IAV in the lower hind limb, or intranasally immunized with 4μg 3R-NC plus 1μg KFD after anesthesia with pentobarbital sodium (50 mg/kg) twice.

**Enzyme-linked immunosorbent assay (ELISA)**

Similar as described previously^1^, 96-well plates were coated with purified recombinant RBD protein (2 μg/mL) in the carbonate-bicarbonate buffer overnight at 4℃, washed with washing buffer (PBS plus % 0.05 Tween-20), blocked with 1% BSA for 2 h at 37℃. Then the samples diluted with serially four-folds were added into wells and incubated at 37℃ for 2 h. After washing, alkaline phosphatase-labeled Goat Anti-Mouse IgG antibody or Goat Anti-Mouse IgA antibody (Southern Biotech) was added 1:4000 (v/v). Followed by washing and substrate (p-nitrophenyl phosphate, Sigma) coloring, the OD405 were read by microplate reader (Thermo Labsystems).

**Pseudotyped virus neutralization assay**

The lentivirus background pseudotyped virus expressing SARS-CoV-2 spike or SARS-CoV-1 spike were generated as previously described ^1^. Briefly, to assess neutralizing efficiency in serum, sera were serially diluted from 1:10 to 1:3200 with DMEM supplemented with 10% FBS. Then 50 μl diluted sera were co-incubated with 20 μl of 200 TCID_50_ SARS-CoV-2 pseudo-typed viruses in 96-well plates at 37℃ for 1h. Next, in each well, 3×10^5^ ACE2-293T cells in 30 μl complete DMEM media were added and incubated at 37℃ with 5% CO_2_ for 48 h.

To assay the neutralizing efficiency in saliva, saliva was firstly inactivated at 56℃ for 30 min, serially diluted from 1:2 to 1:54 with DMEM supplemented with 10% FBS. Then 50 μl diluted samples were co-incubated with 20μl of 200 TCID_50_ SARS-CoV-2 pseudo-typed virus in 96-well plates respectively, at 37℃ for 1 h. Next, 3×10^5^ ACE2-293T cells in 30 μl DMEM complete media were added per well and incubated at 37℃ with 5% CO_2_. After 18 h incubation, cell culture supernatant was changes by new cell culture medium. Then the ACE2-293T cells were further incubated at 37℃ for 30 h with 5% CO_2_.

At last, luciferase activity was analyzed using the luciferase assay system (Promega). Then the inhibition of SARS-CoV-2 pseudotyped virus was calculated, and 50% neutralization titers (NT_50_) were determined as previously described ^2^, by the four-parameter logistic regression in the GraphPad Prism 8.0.

**Preparation and inoculation of SARS-CoV-2 Omicron variant**

For protection experiment, vaccinated hACE2 mice were handled in biosafety level 3 animal facilities (ABSL-3) in accordance with the recommendations for animal care. Mice were intranasally inoculated with 50 μl DMEM containing 5×10^4^ TCID_50_ of SARS-CoV-2 Omicron strain BA.1 per mouse under proper anesthesia, and efforts were made to minimize any potential pain and distress. One of seven mice in the IAV boost group died accidentally at the time of inoculation. Other mice of all four groups were euthanized at 3 days post-infection. Tissues including the lung and turbinate were harvested.

**Histology**

The lung samples from mice were ﬁxed in 4% paraformaldehyde at room temperature for 1 week, embedded in paraffin, sectioned, stained by hematoxylin & eosin (H&E), and imaged by slide scanner Pannoramic MIDI (3DHISTECH) as previously described ^1^. Pathological changes were scored on a 0-5 severity scale, based on thickened alveolar walls and cell inﬁltration. The infiltration was evaluated as described previously and scored on a 1-4 severity scale ^1,3^.

**Statistical analysis**

Analyses were performed using the GraphPad Prism 8.0 software. The test of significance was applied as indicated in the figure legends. For correlation analysis, the method of simple linear regression was used. *P* < 0.05 was considered significant. Significance values are indicated as: ns not significant, **P* < 0.05, ***P*< 0.01, and ****P* < 0.001.

**Supplementary References**

1 Yao, Y. F. *et al.* Protective Efficacy of Inactivated Vaccine against SARS-CoV-2 Infection in Mice and Non-Human Primates. *Virol Sin* **36**, 879-889 (2021).

2 He, B. *et al.* Rapid isolation and immune profiling of SARS-CoV-2 specific memory B cell in convalescent COVID-19 patients via LIBRA-seq. *Signal Transduct Target Ther* **6**, 195 (2021).

3 Knudson, C. J., Hartwig, S. M., Meyerholz, D. K. & Varga, S. M. RSV vaccine-enhanced disease is orchestrated by the combined actions of distinct CD4 T cell subsets. *PLoS pathog* **11**, e1004757 (2015).


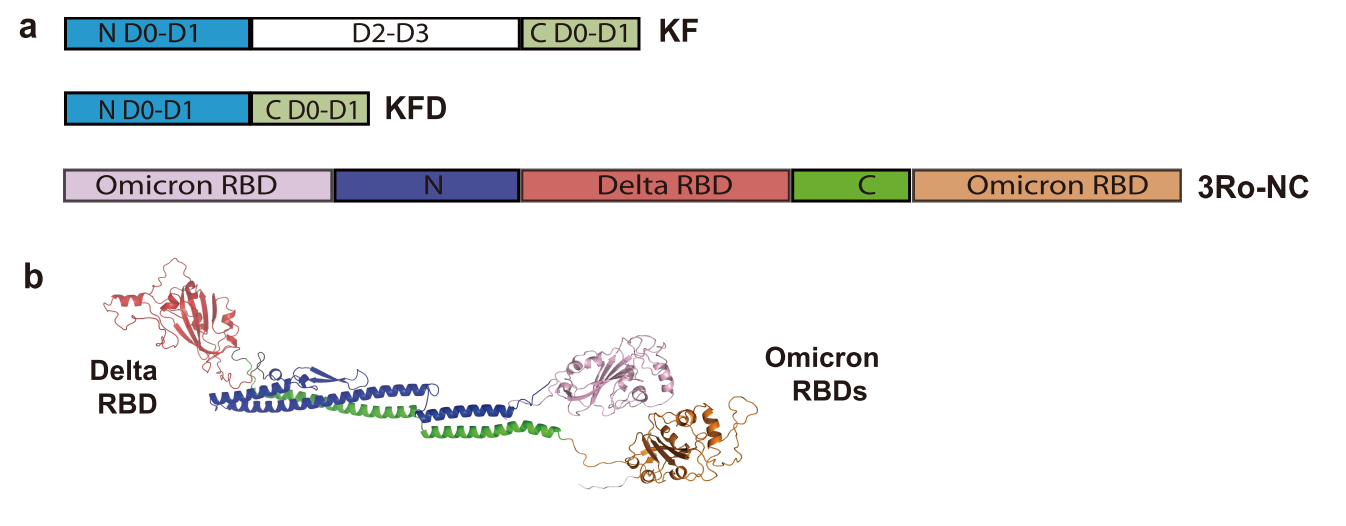


**Figure S1. Construction of the chimeric triple-RBD protein 3Ro-NC.** (**a**) The schematic diagram of recombinant flagellin KF, D0 and D1 domains of KF derived protein KFD, and chimeric protein 3Ro-NC. The scaffold NC was designed according to the 3D structure of KFD. 3Ro-NC gene was generated by connecting three RBD gene of SARS-CoV-2 Delta strain and Omicron BA.1 strain. (**b**) Alpha Fold 2 predicted 3D structure of the protein 3Ro-NC.


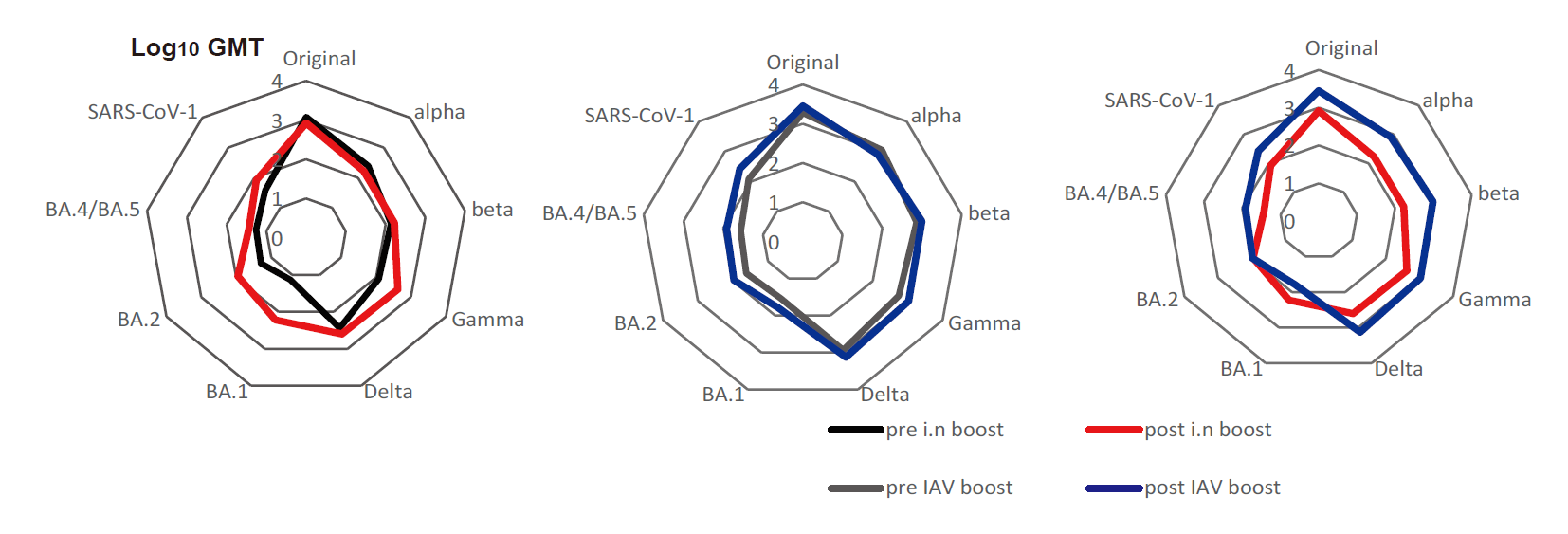


**Figure S2. Geometric mean titers (GMT) of neutralizing antibody before and post the boost** (Related to Figure 1). The 50% neutralizing antibody titers (NT_50_), against different variants of pseudo-typed virus, in serum of the ***i.n boost group*** and ***IAV boost group*** were assayed, before and post the boost (D35 and D77). (**Left panel**), ***i.n boost group*** before and post the boost; (**Middle panel**), ***IAV boost group*** before and post the boost; (**Right panel**), ***i.n boost group*** vs. ***IAV boost group*** post the boost.


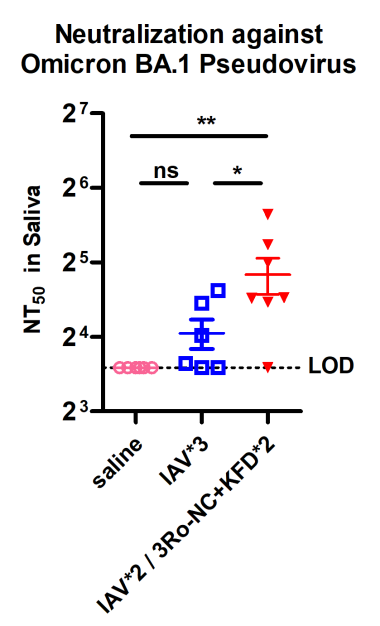


**Figure S3. Neutralizing antibodies in Saliva post boost** (Related to Figure 1)**.** The 50% neutralizing antibody titers (NT_50_) in saliva of the ***i.n boost group*** and the ***IAV boost group*** post boost were assayed post boost (D77). Data are represented as mean ± SEM. Groups were compared using one-way ANOVA. ns, not signiﬁcant; **P* < 0.05; ** *P* < 0.01. LOD, limited of detection.


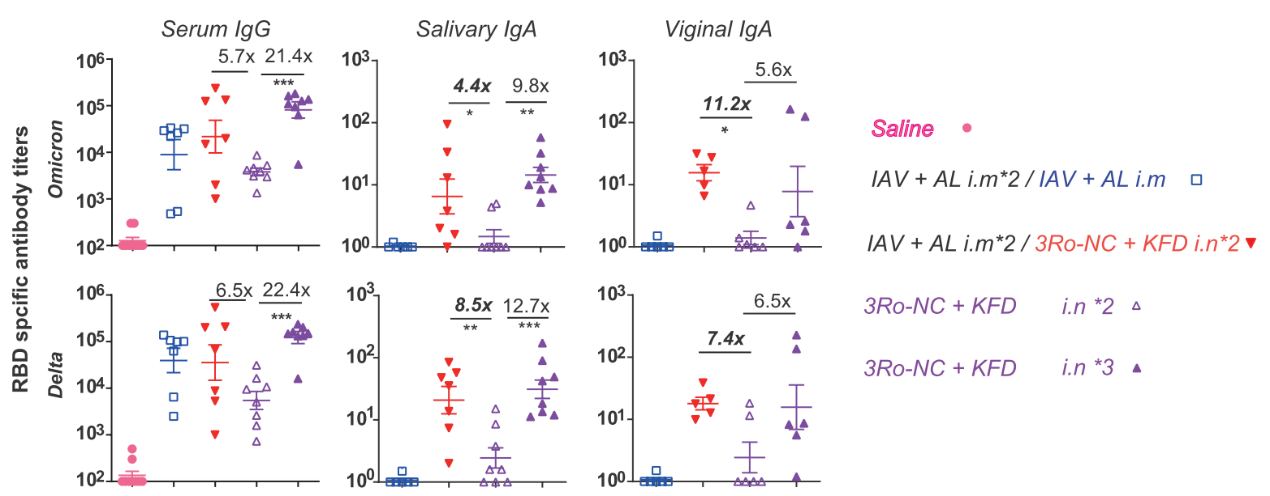


**Figure S4. Omicron BA.1 and Delta strain RBD-specific serum IgG, salivary IgA and vaginal IgA post the final immunization at D77** (Related to Figure 1). Besides the immunized hACE2 transgenic mice shown in Fig.1, two other groups of mice were intranasally immunized with 2 or 3 doses of 3Ro-NC plus KFD (8 mice per group) and compared with the unimmunized saline group (8 mice), ***i.n boost group*** (7 mice) and ***IAV boost group*** (6 mice). Data are represented as mean ± SEM. Groups were compared using one-way ANOVA. **P* < 0.05; ** *P* < 0.01; *** *P* < 0.001.


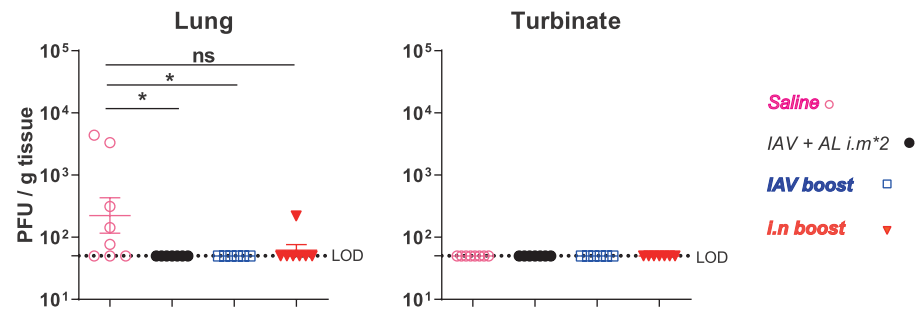


**Figure S5.**  **Plaque assays of Virus loads in the Lung and turbinate of mice at day 3 post SARS-CoV-2 Omicron BA.1 challenge** (Related to Figure 1d and e). Data are represented as mean ± SEM. Groups were compared using one-way ANOVA. ns, not signiﬁcant; **P* < 0.05. LOD, limited of detection.
